# Analysis of the Wear of Forming Tools in the Process of Extruding Ceramic Bands Using Selected Research Methods for Evaluating Operational Durability

**DOI:** 10.3390/ma18091994

**Published:** 2025-04-28

**Authors:** Marek Hawryluk, Jan Marzec, Tadeusz Leśniewski, Justyna Krawczyk, Łukasz Madej, Konrad Perzyński

**Affiliations:** 1 Department of Metal Forming, Welding and Metrology, Wroclaw University of Science and Technology, Wybrzeże Wyspiańskiego 27, 50-370 Wroclaw, Poland; jan.marzec@pwr.edu.pl (J.M.); tadeusz.lesniewski@pwr.edu.pl (T.L.); justyna.krawczyk@pwr.edu.pl (J.K.); 2 Department of Applied Computer Science and Modeling, AGH University of Krakow, Al. Mickiewicza 30, 30-059 Kraków, Poland; lmadej@agh.edu.pl (Ł.M.); kperzyns@agh.edu.pl (K.P.)

**Keywords:** durability of forming tools, extrusion band process, tribological wear, abrasion resistant steel, numerical modelling (mesh-free SPH)

## Abstract

This article presents the results of research concerning a comprehensive analysis of the operation of tools used for forming ceramic roof tiles in the clay-based band extrusion process. The conducted studies demonstrated that key process parameters, such as extrusion pressure and the flow speed of the ceramic mass containing hard components, are crucial for the durability of the tools, significantly affecting their wear. The analysis of the formed mass revealed the presence of hard fractions, such as quartz, zircon, and garnet, which significantly contribute to tool abrasion. Among the tested hardening variants of NC11LV steel, the best results in terms of enhanced longevity were operational tools treated at 1020 °C and then tempered at 200 °C for two hours. These results were confirmed in both operational tests and the dry abrasion test, indicating high wear resistance. Additional hardening through nitriding further extended the tool’s lifespan. The greatest wear was observed in the tool made of Hardox 600 steel with an additional overlay weld, which was caused by improper welding techniques. Numerical modeling, particularly the mesh-free SPH approach, proved to be the most effective method for analyzing the ceramic mass extrusion process.

## 1. Introduction

The ceramic industry belongs to the heavy industry sector, where production utilizes various natural materials based on silicates, as well as clay and other substances. One of the main branches of this industry is the production of construction ceramics, which primarily includes high-durability ceramic tiles and bricks with various applications, as well as a wide range of ceramic roof tiles, commonly used for roofing. Clay, the main component of the material used for roof tiles, is formed through the accumulation of sediments, mainly quartz, feldspar, and clay minerals [1]. Among other roofing materials (such as metal roofing sheets, bitumen shingles, and concrete tiles), ceramic roof tiles stand out as the most optimal option due to their versatile properties and favorable quality-to-price ratio [2,3]. They are characterized by long service life, good acoustic insulation, and the ability to be used on roofs with minimal slope, making them an ideal solution for various types of buildings. Manufactured from natural raw materials, including clay with additives, they are an environmentally friendly solution. Their versatility contributes to the growth of the ceramic industry, opening new possibilities in design and architecture.

The fundamental aspect of the development of the ceramic industry in the field of ceramic roof tile production is improving process efficiency, which is primarily related to the materials used for machine components and equipment for processing and manufacturing ceramic products. On the one hand, efforts focus on finding solutions for the use of new or alternative materials for such machine components, while, on the other hand, the goal is to extend their operational lifespan [4,5]. This is mainly driven by economic factors, increasing production efficiency, and, most importantly, growing environmental requirements and the need to reduce pollution of the natural environment [6,7,8]. Machine components, particularly the forming tools used to shape the clay strand in the extrusion process, are especially susceptible to tribological wear due to intense material flow and friction with the processed ceramic mass. This mass consists of clay (70%), quartz sand (10%), recycled ceramic powder (18%), and other materials [9,10,11]. An essential additive influencing the ability to shape the material during extrusion is water. The water content affects the density, plasticity, and cohesion of the extruded material [12]. Intense abrasive wear is primarily caused by hard fractions within the processed mass, which function as abrasives and lead to tool degradation. It is also important to mention that high friction increases the contact temperature, which can further intensify the wear processes of the forming tools, especially on the surface layer. The movement of abrasive particles during the extrusion of the clay strand can result in scratching, micro-cutting, or plastic deformation (grooving) of the tool material [13]. The most intense abrasion occurs in the contact zone between the extruded ceramic mass and the working surfaces of the forming tools. To ensure a sufficiently long service life of these tools, wear-resistant materials are used. These primarily include Hardox wear-resistant steels and cold-work tool steels, as well as alternative non-metallic materials such as hard ceramics and composite materials [14,15]. In the case of cold-work tool steels, NC11LV (X153CrMoV12) is particularly noteworthy when subjected to heat treatment, which ensures high hardness due to the appropriate distribution of carbide precipitates, mainly M_7_C_3_. The size, shape, and dispersion of these carbides significantly contribute to increased hardness, which in turn reduces tribological wear [16,17]. These steels owe their good wear resistance to their high chromium content (10–15%) and alloying additions of molybdenum and vanadium (around 1%). Due to their good machinability, the possibility of applying additional protective coatings, and relatively low cost, they serve as an alternative to Hardox steels, which, despite their higher impact resistance, are more difficult to machine and more expensive. Other modern steel materials are also being considered and studied, including wear-resistant steels (Brinar, XAR, RAEX, and others), as well as boron and manganese steels (Hadfield steel) [18,19]. Other heat-treated cold-work tool steels also exhibit excellent performance characteristics and the possibility of additional thermo-chemical treatment [20,21]. However, Hardox wear-resistant steels, due to their relatively easy weldability, can also undergo surface welding followed by finishing electrical discharge machining (EDM) [22,23]. There are also solutions involving the development of modular or composite tools, where the working part that comes into direct contact with the formed ceramic mass is made of hard and wear-resistant ceramic inserts. The rest of the tool is made of tool steel or standard structural steel, which helps reduce unit production costs. Therefore, a comprehensive analysis of tool performance and the development of advanced technologies for manufacturing clay-forming equipment significantly enhance their durability and efficiency. The issue of tool wear in shaping processes, such as forging and extrusion, is also relevant in other industries, including metallurgy, metal forming, and plastic processing [24,25]. Therefore, the methods for analyzing and evaluating surface quality for most tools used in manufacturing processes are similar and include macroscopic examination, surface scanning, statistical wear analysis, metallographic microscopic studies, microhardness measurements, and assessments of working tools [26,27,28]. All these methods enable a detailed understanding of wear mechanisms and the development of strategies to minimize them. Conducting such analyses is complicated, regardless of the application, due to numerous factors affecting the operational process, including high and intense friction, high and variable pressures, temperature gradients, and long friction paths. Therefore, to support this research, CAD/CAE/CAM techniques and numerical modeling are increasingly used, primarily for design and optimization. By employing numerical simulations, it becomes feasible to pinpoint and evaluate the phenomena and process parameters in extrusion that are hard to detect during machine operation [29,30,31]. Such analyses are also conducted in the ceramic industry, but current tool degradation models are not yet fully developed analytically [32,33]. However, effective modeling can significantly contribute to increasing tool durability and reducing production costs. As a result, higher product quality can be achieved, which directly impacts customer satisfaction and market competitiveness. Further research and development in this area could lead to the discovery of new solutions that will further revolutionize the ceramic industry and contribute to its sustainable development.

## 2. Materials and Methods

The main focus of the conducted research is the analysis of the tools used to form ceramic roof tiles in the band extrusion process. During this stage, the mass is compressed and shifts at the highest rate, leading to particularly intensive degradation of the forming tools. These elements are responsible for shaping the material, determining its thickness, shape and surface quality. Therefore, the proper design and selection of the forming tool material have a crucial effect on the quality of the formed band and, consequently, the final product. The first step of the studies involved a comprehensive analysis of the technology, particularly focusing on the key process parameters and the characteristics of the formed mass. To that end, the following studies were conducted:

- Measurements of the extrusion pressure by VEGABAR 38 system (WEGA, Schiltach, Germany;
- Thermovisual analysis by thermovision camera Flir 840 (FLIR Thermal Studio Starter, Teledyne FLIR LLC, 27700 SW Parkway Avenue, Wilsonville, OR, USA);
- Hardness measurements were carried out with the use of the Vickers method by means of a Leco AMH55 hardness tester (Leco Corporation, St. Joseph, MI, USA).
- Measurements of the band velocity by LMS Laser Speed Sensor (RHEINTACHO Messtechnik, Freiburg, Germany);
- SEM-EDS (Quanta 650 FEG Scanning Electron Microscope, ThermoFisher Scientific, Waltham, MA, USA) and XRD (Empyrean X-ray diffractometer, Malyern Panalytical, Malvern, Worcestershire, UK) analysis of the formed mass;
- 3D scanning of geometric changes of tools (optical scanner GOM ATOS II, GOM, Braunschweig, Germany); A compression test of the formed mass (own work, Wrocław University of Science and Technology, Wroclaw, Poland);
- Numerical modelling by use Abaqus 2024 (Simulia, Johnston, RI, USA)

Next, further analyses included operational tests under production conditions of forming tools, which were additionally protected against wear. To that end, materials were selected and properly prepared according to Table 1, with the standard tools used in the process (Forming Tool 2) serving as the reference point to compare the obtained results.

The operational tests of the forming tools were conducted on a two-band pug mill in a standard process of ceramic roof tile production on an industrial scale. The two-band extruder made it possible to form tow bands with the same parameters simultaneously, which ensured similar working conditions in the case of both tools. The machine used to perform the tests generated an extrusion pressure of 19–21 bar, which translated to an average band velocity at the level of 20 m/min. Tests for individual tool variants were conducted with an intended operating time of 170 h. According to the data in Table 1, the reference point for each applied durability improvement variant in the operational tests was Forming Tool 2, made of NC11LV steel hardened at 1020 °C and tempered at 200 °C for 2 h. Comparisons of the various material variants were carried out using the 3D scanning technology and macroscopic analysis.

An extension of the wear resistance tests for tool materials involved a dry abrasive test, which was conducted on a Tester T-07 test stand built in 2007 (The Institute for Sustainable Technologies in Radom, Poland) This test utilizes loose, dry abrasive material that is fed between the sample and a rotating disc, which operates at a predetermined rotational speed of 1800 rpm for 30 min. The final stage of the research involved the use of numerical modelling to expand the possibilities for analyzing the band forming process for ceramic tiles. This is due to the fact that the industrial band extrusion process itself is a large-scale and complex technology, which presents numerous challenges in determining or defining the key parameters and physical quantities that affect the process efficiency and the operational durability of forming tools. The acquired knowledge was used to develop a numerical model and conduct preliminary simulations using the Abaqus software 2024.

## 3. Results and Discussion

### 3.1. Analysis of Band Extrusion Process Parameters

**Figure 1:**
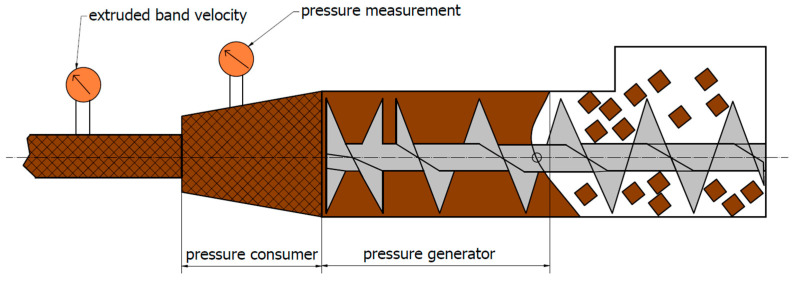
Diagram of the band extrusion process [34].

An important aspect of studying the wear of forming tools is the analysis of operational parameters, which allows for the verification of the key factors affecting the wear mechanisms. The primary stage of the mass forming process for ceramic tiles is the band extrusion process, which is carried out in pug mills. A typical pug mill, shown in Figure 1, is divided into two zones: the compression zone (pressure generator) and the shaping zone (pressure receiver). In the first stage of the process, the mass is degassed in a vacuum chamber and then subjected to compression and forming by screws, which ensure its movement and compression. In the second phase, the mass passes through a set of tools consisting of a conical head, a nozzle and a forming tool (overlay) that shape the material, determining its thickness, shape and surface quality. The mass formed in this way is then cut into appropriate segments and conveyed by roller feeders to the subsequent stages of production. The most important process parameters, including the extrusion pressure and the band velocity, can be recorded by sensors located at different stages of forming.

**Figure 2:**
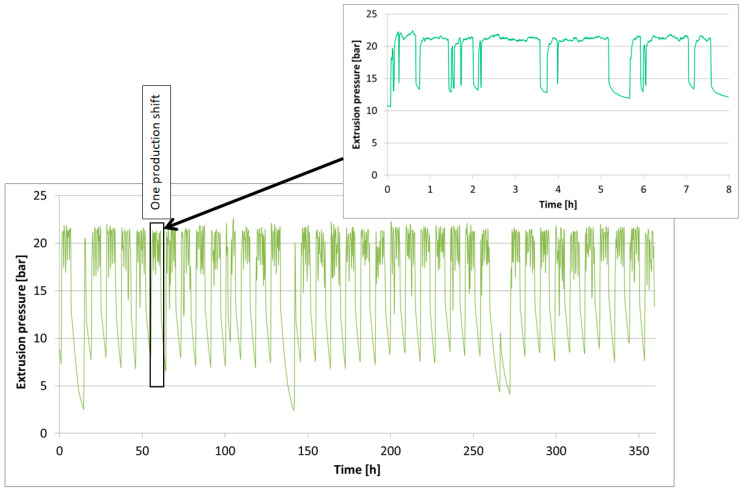
Diagram of the band extrusion pressure.

The tested tools operated for the assumed production cycles on a twin-shaft pug mill with a cylinder diameter of 350 mm and a flow capacity of approximately 15 t/h. The process parameters recorded during this time have been shown in Figure 2 and Figure 4.

In Figure 2, the diagram illustrates the relationship between the extrusion pressure and the operating time. The chart clearly shows periods when the mass is being formed as well as production breaks. Each drop in pressure below 20 bar is caused by machine stoppages for retooling or parameter adjustments at other stages of the process. During extrusion, the pressure remained mostly around 21 bar. The slight pressure fluctuations visible on the diagram result from the operation of the sensor system and the PID controller, which aims to equalize the actual process pressure to the set output value. The high band extrusion pressure intensifies the effect of the hard fractions in the production mass on the forming tool, demonstrating the extreme conditions under which these tools operate.

**Figure 3:**
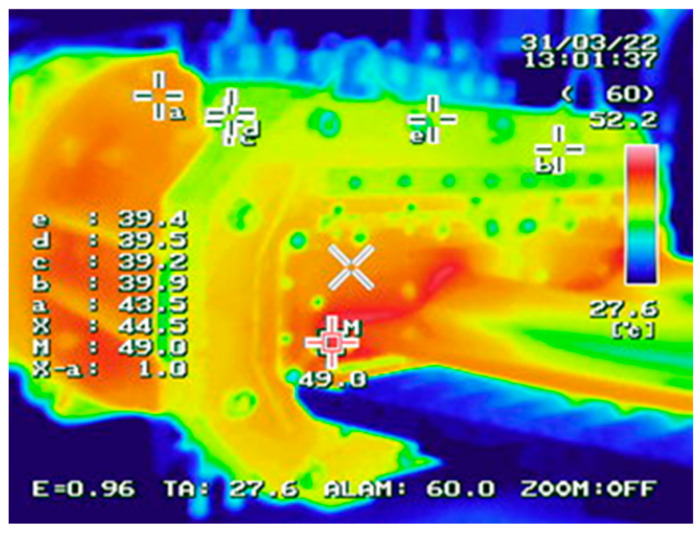
A photograph of the thermal imaging camera.

At the point of direct contact between the forming tools and the mass, high friction occurs, resulting in an increase in temperature, which was recorded using a thermal imaging camera, as shown in Figure 3. The temperature in the contact area between the clay band and the tool ranges from 45 to 50 °C.

**Figure 4:**
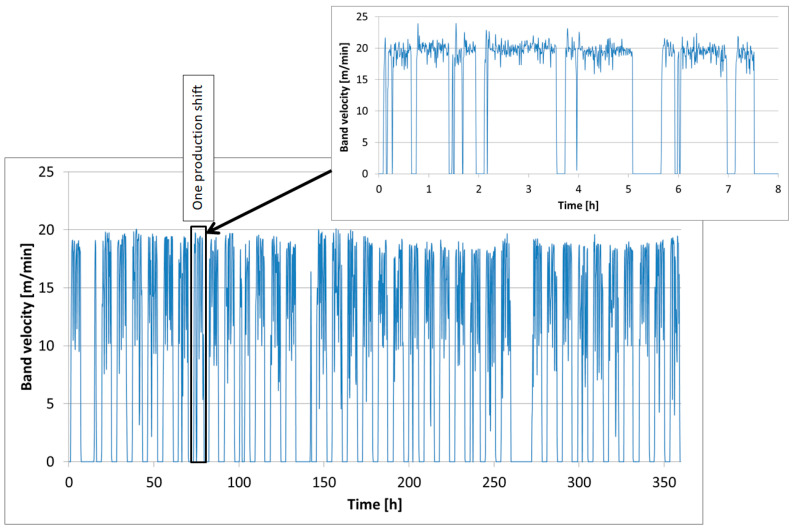
A diagram of the extruded band’s speed.

Another important process parameter recorded during extrusion is the band speed, which is illustrated in the diagram in Figure 4. This measurement is taken after the mass exits the pug mill in the form of a band. The band speed is a result of the operation of the whole forming machine system, and fluctuations in this value are caused by variations in the amount of mass supplied to the process and compressed at a given moment.

As we can see in the detailed diagram, the band speed in the analyzed process fluctuated around 20 m/min. This parameter also directly affects the overall production efficiency, as at this extrusion speed, the twin-screw pug mill enables the production of 70–80 tiles per minute. The points on the diagram where the speed drops to 0 m/min indicate pug mill stoppages. The most common causes of these short downtimes include machine retooling or excessive band speed, which leads to difficulties in cutting the material into the specified segments.

### 3.2. Analysis of the Formed Mass

#### 3.2.1. XRD and SEM-EDS Methods

An important aspect of analyzing the band forming process and the wear mechanisms occurring during this process is understanding the exact characteristics of the formed mass, which abrasively affects the machine components, particularly the forming tools (inserts) that define the final shape of the band. Standard masses used in the production of building ceramics, including ceramic tiles, consist of three main groups of components: plastic materials, tempering agents, and fluxes.

**Figure 5:**
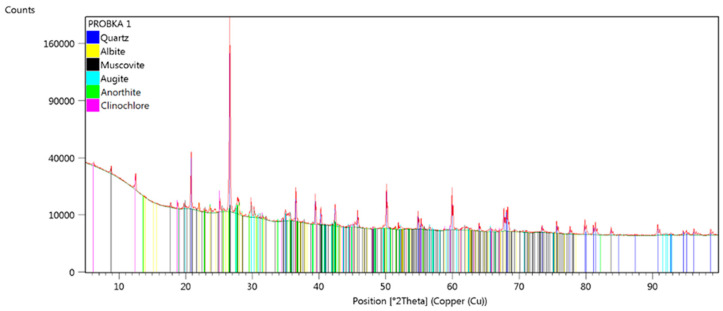
An XRD analysis of the mass for the ceramic roof tile production.

The plastic component used in tile production consists of clay-based raw materials, primarily clays, which naturally possess the ability to form a plastic mass when combined with water. The primary additives are non-plastic materials (tempering agents), including quartz sand, ground brick rubble, and furnace waste, which serve to reduce the plasticity of the mass and, consequently, its shrinkage during drying and firing. The final group of components consists of fluxes, which are added to lower the sintering temperature of the mass and reduce the porosity of the finished products. In terms of their effect on the abrasive wear and the degradation of forming tools, the presence of hard fractions with highly developed surfaces, as well as their size and quantity within the formed mass, plays a crucial role. For a precise identification of the formed mass, an XRD analysis combined with the SEM-EDS method is appropriate. The results of the XRD analysis of the mass have been shown in Figure 5.

The XRD analysis of the mass used for ceramic tile production revealed the presence of the main mineral phases: quartz, plagioclase group (albite-anorthite), augite, clinochlore (chlorite), and muscovite. Despite the concentration of magnetic phases and the separation of the clay fraction, no additional mineral phases could be identified using this method.

**Figure 6:**
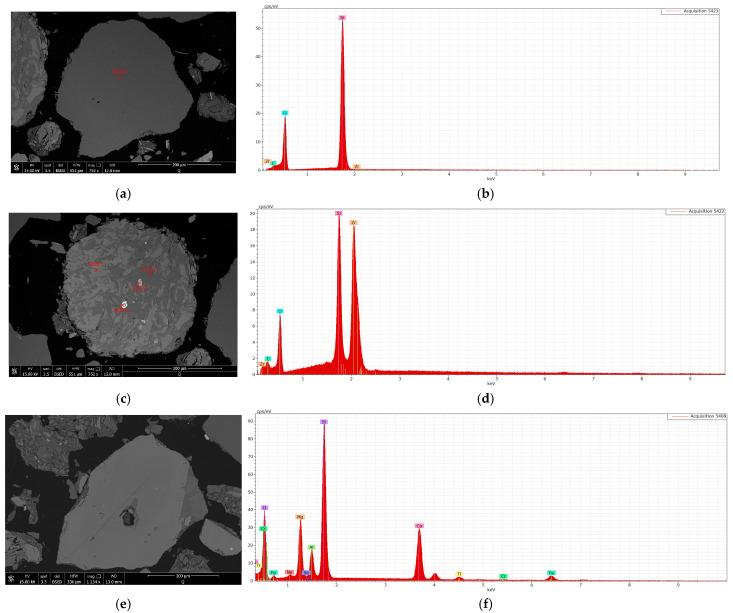
SEM images and an EDS spectrum of the mass used for ceramic tile production: (**a**) quartz SEM, (**b**) quartz EDS, (**c**) zircon SEM, (**d**) zircon EDS, (**e**) garnet SEM, (**f**) garnet EDS.

The SEM-EDS method proved to be the most effective for analyzing this type of samples, allowing for the identification of most phases present in the material. The SEM-EDS analysis was conducted on fractions larger than 4 μm. The samples consisted of grains, mineral aggregates, ceramic fragments, and rock fragments, with mafic rocks (basaltoids) and biotite-muscovite schists predominating. The main components of these schists include muscovite, biotite, and quartz. The key minerals identified as critical for wear have been shown in Figure 6.

Intensive abrasive wear of the forming tools is largely caused by the presence of hard fractions in the formed mass. The conducted studies identified several minerals characterized by high hardness on the Mohs scale. The most abundant of these is quartz (Figure 6a), which has a Mohs hardness of 7. Other high-hardness minerals present in the mass include zircon (Figure 6b) with a Mohs hardness of 6.5–7.5 and garnet (Figure 6c) with a hardness of 7–7.5. Most of these hard fractions originate from tempering additives, which, in the case of the mass used for building ceramics production, include quartz sand and ground basalt grit. These materials contain the identified hard minerals.

**Figure 7:**
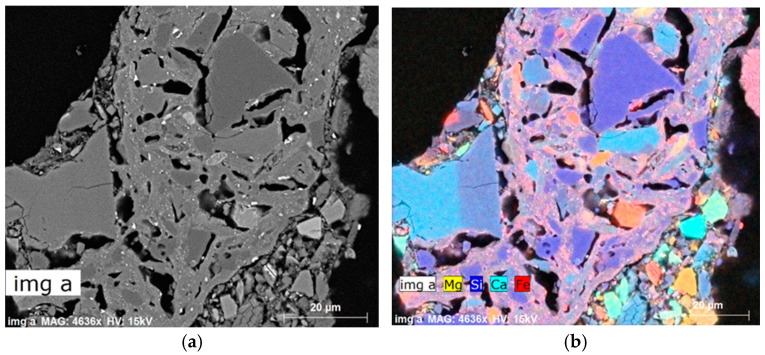
Ceramic fragment in the mass used for ceramic tile production: (**a**) BSE images, (**b**) combined with a map of elements.

The SEM-EDS analysis also detected ceramic additives from recycled materials, which are presented in Figure 7.

A ceramic fragment with characteristic wedge-shaped pores results from the volume change of the material (mainly clay minerals) due to the loss of both free and bound water during the firing process. The presence of ceramics in the mass indicates the use of tempering additives derived from recycled defective production waste. In the case of the industrial ceramic tile manufacturing process, these typically consist of defective products rejected during quality control, which are then ground in ball mills and reintroduced into the process in the form of fine powder. The hardness of these ceramic fragments does not exceed the maximum hardness of their constituent minerals, such as quartz and garnet.

**Figure 8:**
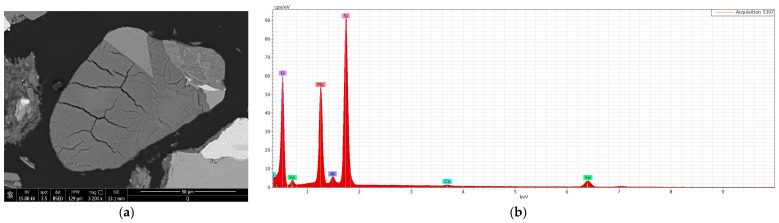
SEM images and an EDS spectrum of the mass used for ceramic tile production clay material (vermiculite): (**a**) SEM, (**b**) EDS.

The only clay mineral Identified In the analysis Is vermiculite (Figure 8); none of the analyses revealed the presence of other clay phases such as illite, kaolinite, montmorillonite, smectite, or glauconite. The presence of vermiculite is well justified, considering the structure of the granular skeleton, which consists of volcanic-origin rocks (basaltoids) and biotite-muscovite schists, as it forms at the cost of biotite mica present in both rock types. It constitutes the primary plastic component of the analyzed mass and, due to its relatively low hardness (1–1.5 on the Mohs scale), it does not significantly contribute to the wear of forming tools. However, its characteristics ensure good plasticity.

#### 3.2.2. Study of Band Material Characteristics—Determination of Flow (Plasticity) Curves

**Figure 9:**
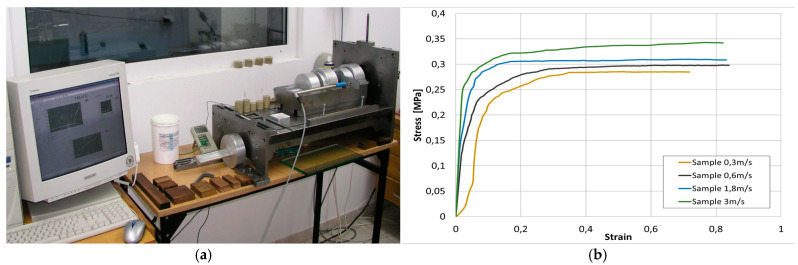
The view of: (**a**) physical modeling press, (**b**) flow curves—stress-strain of clay.

One of the key properties of the mass used for ceramic tile production is its good deformability and plasticity. For relatively soft materials, a suitable method for determining the flow characteristics is the compression (upsetting) test, which provides more detailed information about the material that can be utilized in more precise analyses within numerical simulations. The samples for these tests were prepared through extrusion under laboratory conditions, with the parameters corresponding to the production conditions. The upsetting test was then conducted using a specialized press for physical modelling. Based on the obtained results, the flow stress curves were determined and have been presented in Figure 9.

The conducted tests for determining the stress-strain curves revealed that the mass used for ceramic tile production is sensitive to changes in the deformation speed. This behavior is typical for materials undergoing plastic deformation under warm and hot conditions. As the deformation rate increases, the stress value also rises. However, no material weakening was observed, which is a typical phenomenon during the hot deformation of metals. For the baseline speed of 0.3 m/s, which is the closest to the band forming conditions in the production process, the yield stress is 0.28 MPa, and the strain reaches 0.85. At higher speeds, the stress systematically increased, reaching 0.34 Mpa at the maximum analyzed speed of 3 m/s, while the strain slightly exceeded 0.8. However, at a speed of 4 m/s, the sample experienced rapid failure, making accurate force measurements difficult and potentially leading to errors in further studies.

Based on the determined characteristics, they can be described using an appropriate rheological model, which can then be applied in numerical modelling of the extrusion process. A well-defined rheological model will undoubtedly facilitate numerical simulations within the selected computational package. Preliminary studies conducted by the authors have shown that the behavior of the band material, based on clay with additives, can be fairly well described using Spittel’s equation [29]. The initial attempts to describe the rheology of the extruded material using the HS (Hans Spittel formula) model are presented as follows:

(1) $$\sigma_{f}=A\varepsilon^{m_{2}}\exp\left(\frac{m_{4}}{\varepsilon }\right){\dot{\varepsilon }}^{m_{3}}$$

where:

ε—equivalent strain;

$\dot{\varepsilon }$—equivalent eformation rate;

*A*, *m*_2_, *m*_3_, *m*_4_—model coefficients dependent on the material.

**Figure 10:**
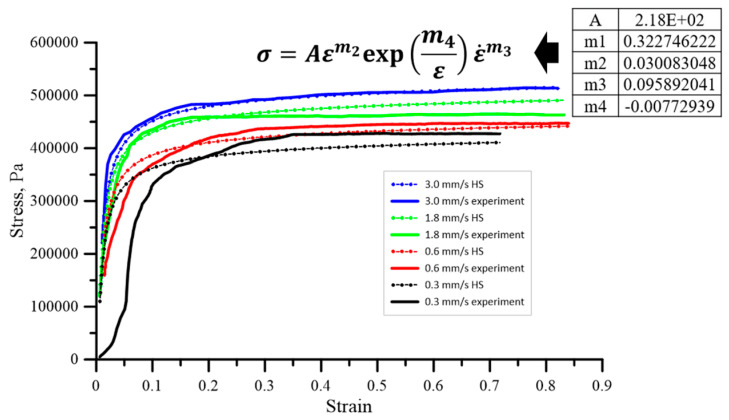
The HS equation coefficients to the scope of the measurement data [32].

The model coefficients were determined based on the inverse analysis concept. The identified values of the HS equation, along with the corresponding comparisons between the predicted and experimental flow stress curves, are presented in Figure 10.

Due to its specific nature, the Hansel-Spittel model is well-suited for representing materials in plastic forming processes, including the extrusion of bands for ceramic tiles. The selected model allows for the depiction of the relationship between flow stress, strain, strain rate, and temperature. This prepared model can then be utilized in numerical simulations, which will be best conducted using the Abaqus software.

### 3.3. Operational Analysis of Forming Tools

The durability of the forming elements for ceramic tile bands is especially affected by the properties of the surface layer, as it is the first to come into contact with the formed mass. For this reason, placing particular emphasis on surface modifications of the tool should bring the greatest effects in terms of extending their service life.

#### 3.3.1. Hardness Analysis

**Figure 11:**
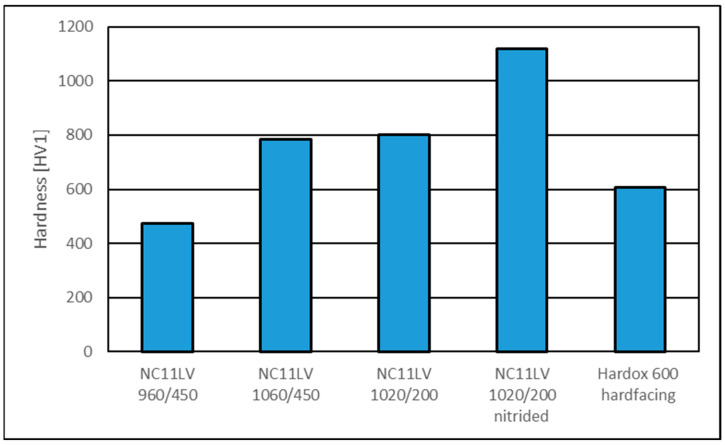
Results of hardness measurements of tested materials.

In the first stage of the operational analysis, the hardness of the surface layer of the tools made from the analyzed materials was examined. The obtained results are shown in Figure 11.

The hardness of the tools made from hardened steel varied depending on the applied heat treatment. The lowest hardness, measuring 475 HV1, was observed in the sample hardened at 960 °C and tempered at 450 °C. The remaining samples had higher hardness values of 786 HV1 (hardened at 1060 °C and tempered at 450 °C) and 802 HV1 (hardened at 1020 °C and tempered at 200 °C). For the tool that underwent additional nitriding, the surface hardness reached 1120 HV1. In the case of the layer welded onto the surface of the Hardox steel tool, the hardness was 606 HV1.

#### 3.3.2. Geometric Analysis—3D Scanning

A key step in evaluating methods to extend the durability of materials used for forming tools for ceramic tile bands was the operational testing. The wear studies employed geometric analysis of the wear on the lower forming tools based on 3D scanning.

##### Geometric Analysis—3D Scanning

The first stage of the conducted operational tests on ceramic tile forming tools involved the study of tools made from NC11LV steel, which underwent various thermal treatment variants. The variants being tested were:

- Test 1—Steel NC11LV—hardening: 960 °C, tempering: 450 °C for 2 h
- Test 2—Steel NC11LV—hardening: 1060 °C, tempering: 450 °C for 2 h

**Figure 12:**
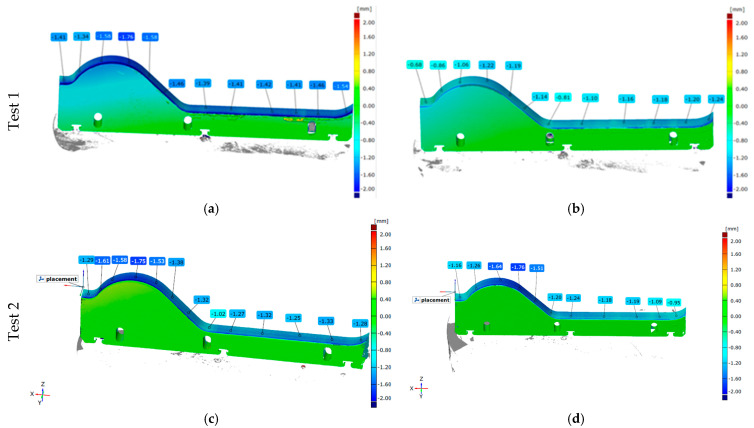
Results of the scanning of the lower forming tools: (**a**,**b**) Test 1 and (**c**,**d**) Test 2.

Both thermal treatment variants underwent their production cycles alongside tools made from NC11LV steel, which were hardened at 1020 °C and tempered at 200 °C. The results obtained from this part of the study have been shown in Figure 12.

Analyzing the scanning results for different thermal treatment variants of the lower forming tools, it can be concluded that the material loss is generally uniform along the entire shaping line. More intense wear can be observed on the side where the material enters the tool. In all the tested tools, it can also be observed that slightly higher wear occurs in the shaping area, commonly referred to as the “wave”. It can be noted that, across the whole working surface, the greatest material loss occurs for NC11LV steel hardened at 960 °C and tempered at 450 °C. For this variant, the wear level on a significant portion of the working surface exceeded 1.40 mm, reaching a maximum of 1.76 mm. In a direct comparison within Test 1, the NC11LV steel hardened at 1020 °C and tempered at 200 °C demonstrated better wear resistance, with the maximum wear level reaching 1.22 mm, and in some areas, the wear did not exceed 1 mm. In contrast, Test 2, where this thermal treatment variant of NC11LV steel was compared with a tool hardened at 1060 °C and tempered at 450 °C, showed that the wear levels were comparable. The average wear for both samples ranged from 1.00 to 1.75 mm.

##### Test of Tools Made of NC11LV Hardened Steel, Additionally Nitrided

**Figure 13:**
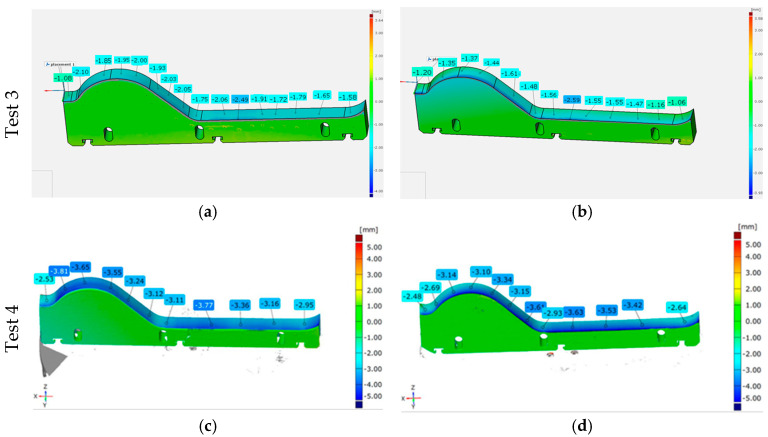
Results of the scanning of the lower forming tools: (**a**,**b**) Test 3 and (**c**,**d**) Test 4.

As part of the next stage of operational tests, the effect of the nitrided layer on the durability of forming tools in the extrusion process of the band for ceramic tiles was examined. The tests were conducted in two stages, during which each tool worked for approximately 170 h. The complete results of the 3D scanning are presented in Figure 13. In the industrial process, the mass is extruded from a single chamber through two nozzles equipped with geometrically identical tools. Therefore, on the right side of the analyzed scanning results (Figure 13b), there is a standard tool that serves as a reference for other tool variants.

Analyzing the scanning results after 170 h, it can be observed that the additionally nitrided tool exhibited less wear, with most measurements showing wear around 1.50 mm, whereas the wear level of the tool that was only hardened ranged between 1.60 and 2.05 mm. After the second stage, which is 340 h of operation in total, the wear level for both tools increased proportionally to the working time. While the difference in wear still remains at around 0.50 mm in some areas, in most measurement points, the values are now more similar for both materials. This may be due to the wear of the nitrided layer, which led to increased wear in the second stage of the test.

##### Test of the Forming Tools—Hardox 600 Overlay Welded

**Figure 14:**
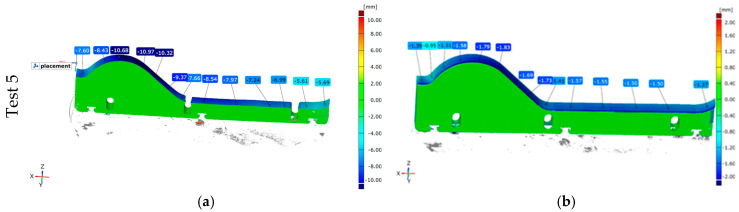
Results of the scanning of the lower forming tools—Test 5: (**a**) surfacing Hardox 600, (**b**) NC11LV hardening 1020 °C and tempering 200 °C.

The final stage of the operational tests involved evaluating a forming tool made of wear-resistant Hardox 600 steel with an additional overlay welded layer under production conditions. The assumption was that this would significantly extend the service life and allow for the regeneration of the surface layer. The results of this trial have been presented in Figure 14.

Despite the use of wear-resistant Hardox 600 steel as the base material combined with an overlay weld with a hardness 10–20% higher than the parent material, the tool experienced critical wear, as shown in the scan in Figure 14a. The wear in the forming area, known as the “wave”, exceeded 10 mm, reaching a maximum of 10.97 mm in some places. The overall wear in the remaining working area of the tool ranged between 5.60 and 8.50 mm, leading to abrasion down to the adjustment holes. None of the other tested tools exhibited such extensive wear. The probable cause was an improperly executed overlay welding process. Despite forming a hard welded layer, the base Hardox 600 steel may have undergone tempering during the process, significantly reducing its wear resistance. Once the overlay layer was worn off, further abrasion occurred at an accelerated rate, leading to critical degradation during the 170-h test. In comparison, the reference tool, made of tool steel hardened at 1020 °C and tempered at 200 °C, wore down to an average of only 1.50 mm over the same period.

**Figure 15:**
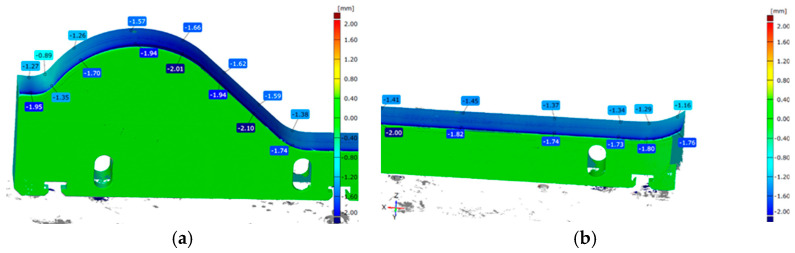
Detailed results of the scanning of the lower tools: (**a**) left part, (**b**) right part.

Figure 15 shows more detailed scanning results. They clearly demonstrate that the tool does not wear uniformly across its entire thickness. The greatest wear appears along the entire length of the tool on the side where the mass enters during the extrusion process, and it decreases in the direction of the mass flow.

#### 3.3.3. Macroscopic Analysis

**Figure 16:**
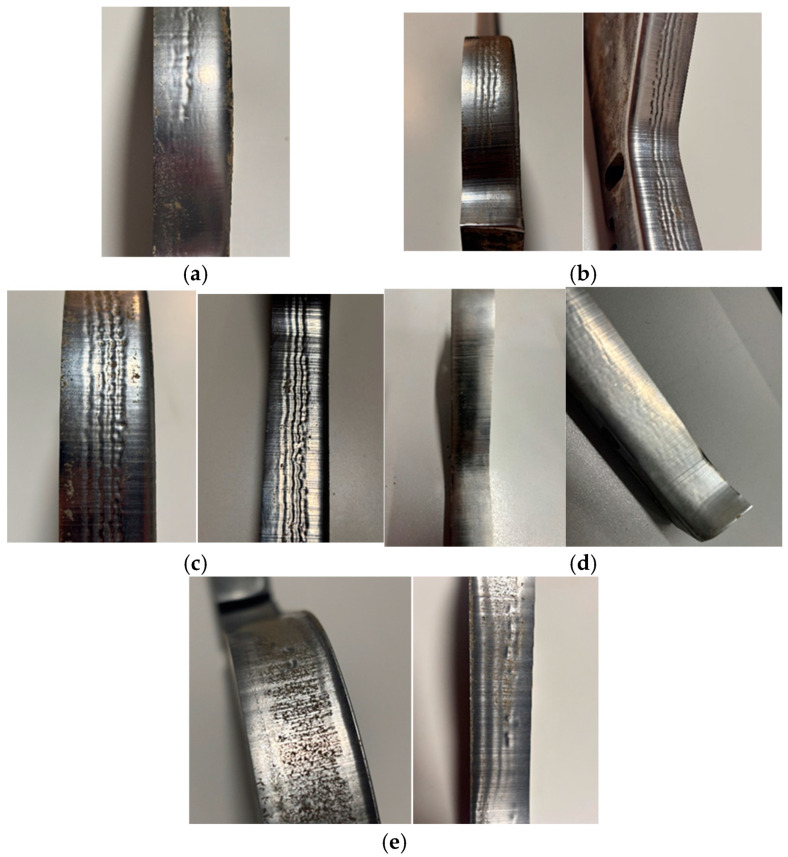
A comparative macroscopic analysis of selected shaping inserts for the clay band: (**a**) 960/450, (**b**) 1060/450, (**c**) 1020/200, (**d**) 1020/200 nitrogen, (**e**) Hardox 600 overlay welded.

A more precise method for verifying the phenomena occurring during the operation of forming tools for ceramic tiles is the macroscopic analysis of the working surface. In the case of the conducted operational tests, this analysis allows for the identification of the wear mechanisms affecting the materials due to contact with the processed mass. Defects that develop on the working surface of the forming tool can lead to imperfections on the surface of the extruded band, which may later become critical for the final product. Figure 16 presents all the forming tools used in the operational tests.

The presented results, in the form of images of the working surfaces from the macroscopic analysis, illustrate the scale of surface degradation for all the tools after 170 h of operation. On the 1020/200 tool (Figure 16c), distinct and regular surface deformations can be observed, forming perpendicular to the direction of the material flow. A similar phenomenon, though to a slightly lesser extent, is also visible on the 1060/450 tool (Figure 16b). For the 960/450 tool, a comparable wear mechanism is significantly less visible and does not maintain continuity along the entire length of the tool. Additionally, on all the hardened tools (Figure 16a–c), scratches aligned with the material flow direction can be observed, resulting from the abrasive particles present in the forming mass. These scratches are most prominent on the 1020/200 (Figure 16c) and 1060/450 (Figure 16b) tools. Other wear phenomena can be observed on the remaining two tools. On the surface of the additionally nitrided 1020/200 tool (Figure 16d), only shallow scratches are visible, which are unlikely to negatively impact the formed band. In contrast, the tool made of Hardox 600 steel with an additional overlay weld (Figure 16e), which experienced the most intensive wear, exhibits numerous scratches and plastic deformations of the material. Additionally, in this tool, a clear flange has formed at the point where the forming mass exits the tool.

**Figure 17:**
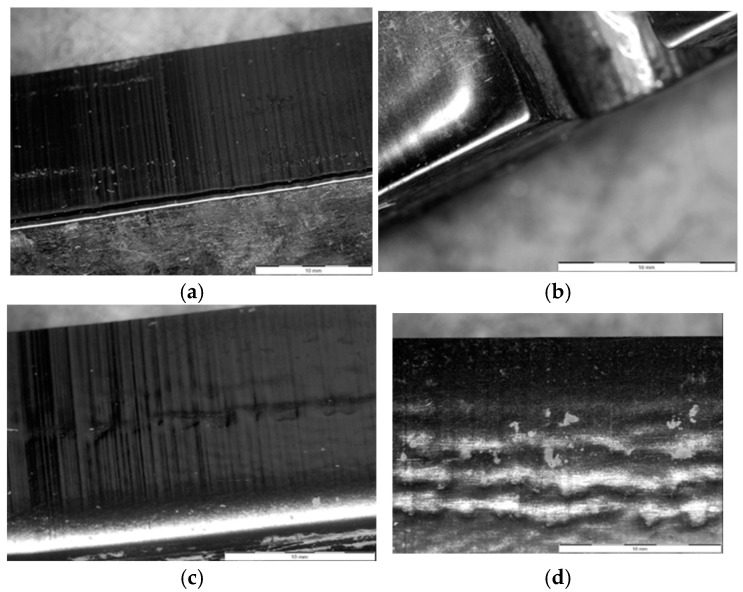
Topography images of the surface taken with a stereoscopic microscope: (**a**,**b**) Hardox 600 overlay welded, (**c**,**d**) NC11LV hardened. Scale bar: 10 mm.

A more detailed macroscopic analysis can be conducted by capturing high-magnification images using a stereoscopic microscope. The images of the working surfaces of the analyzed tools, taken with such a microscope, have been presented in Figure 17.

The images in Figure 17 provide a much clearer view of how the tool surfaces have worn. In both materials, numerous regular scratches on the working surface of the tools are clearly visible. For the hardened NC11LV steel (1020/200), the observed deformations (Figure 17c,d) appear as grooves running along the entire length of the tool. In contrast, the stereoscopic microscope images clearly show the formation of a flange due to wear in the case of the tool made of overlay-welded Hardox 600 steel (Figure 17a,b).

#### 3.3.4. Dry Abrasion Test

A good laboratory method that can serve as an additional verification of the wear resistance of the tested materials is the dry abrasion test. This test provides a good reference point for tool wear in the ceramic forming process due to the type of the used abradant. The abradant in this method is electrocorundum No. 90, which has a hardness of 9 on the Mohs scale. During the test, the abradant is fed between the sample which is pressed with a specified force against a rotating rubber disc with a diameter of d = 50 mm at a predetermined rotational speed. The reference sample in these tests was made of normalized C45 steel. The test result is the weight loss of the sample (the difference in weight before and after the test) after a set friction time (a specified number of rubber roll rotations). Based on these measurements, the relative wear resistance K_b_ can be calculated using the following formula:

(2) $$K_{b}=\frac{Z_{ww}\cdot \rho_{b}\cdot N_{b}}{Z_{wb}\cdot \rho_{w}\cdot N_{w}}$$

where:

Z_ww—_weight loss of the reference sample (g)

Z_wb_—weight loss of the tested material (g)

ρ_w_—density of the reference material

ρ_b_—density of the tested material

N_w_—number of rotations of the reference sample’s friction path,

N_b_—number of rotations of the tested sample’s friction path.

**Figure 18:**
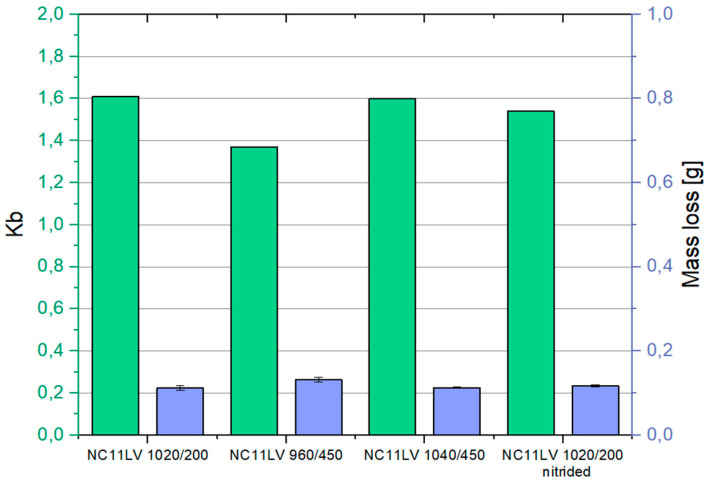
A comparison of the dry abrasion tests results: weight loss—average value [g] and relative abrasive wear resistance K_b_.

The results of the dry abrasion test have been presented in Figure 18. In this test, samples cut from tools made of hardened NC11LV tool steel were examined. The reference sample, in accordance with the standard, was made of C45 steel and tested at 600 rpm. Its average weight loss was 0.062 g.

Analyzing the results of the wear resistance tests using the T-07 tester, it can be observed that the outcomes for hardened NC11LV tool steel are consistent with the operational tests. The NC11LV 1020/200 sample demonstrated the highest wear resistance in this test, with a relative wear resistance parameter K_b_ of 1.646. A similar level of wear was observed for the NC11LV 1060/450 sample, where the K_b_ coefficient reached 1.597. Similarly, the sample NC11LV 1020/200 nitrided tool steel wore out, with relative abrasive wear resistance K_b_ amounting to 1.541. The highest wear, as in the operational test, was recorded for the NC11LV 960/450 sample, with a K_b_ value of 1.393.

### 3.4. Numerical Modelling

Numerous research studies conducted by the authors have shown that for a comprehensive analysis of the operational durability of tools, further investigations should be expanded to include numerical modelling. This approach allows for the definition of various parameters and phenomena that are difficult or even impossible to measure using conventional methods, particularly in determining the key technological parameters of the process, such as tool pressures, forces, and other critical factors [5,32].

The basis for numerical modelling of the extrusion process is the proper definition of the formed mass. This is achieved using the knowledge gained from previous composition analyses and determined material characteristics. In particular, accurate material characteristics, together with their rheological model, are crucial for developing a reliable numerical model.

**Figure 19:**
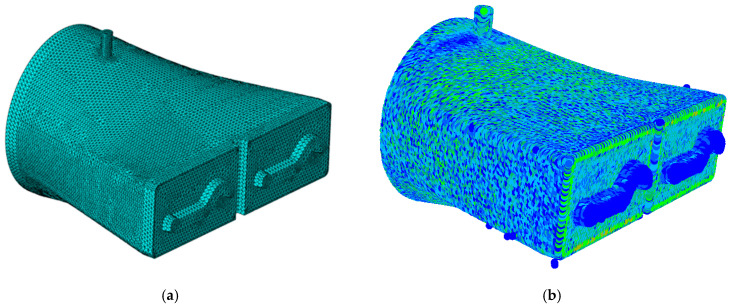
Preliminary models of the forming zone for ceramic tile bands (**a**) MES, (**b**) SPH.

Preliminary research conducted by the authors has shown that the numerical modelling of the extrusion process can be approached in two ways: using a mesh-based method (FEM) or a mesh-free method (SPH), as illustrated in Figure 19. The numerical simulations were performed using the Abaqus software. However, more advanced studies have indicated that accurately modelling such a process, which involves large deformations and mesh distortions of the extruded material, makes the use of FEM challenging due to numerous “numerical” difficulties.

Therefore, the mesh-free SPH approach (Figure 19b) is an interesting method as it eliminates the issue of mesh degradation that occurs in the finite element method (FEM) (Figure 19a). The SPH method is significantly more effective in simulations involving large deformations, such as the forming of ceramic tile bands.

**Figure 20:**
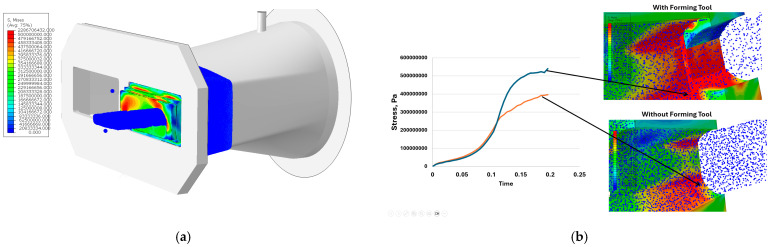
The simulation results of band extrusion: (**a**) distribution of equivalent stresses (von Mises), (**b**) stress vector courses over time at selected tool points.

Based on the SPH mesh-free model of the clay-based forming material used for ceramic tile production, a simulation was successfully conducted, with the results presented in Figure 20. These results enable the determination of parameters that are difficult to measure using other methods.

The results obtained from the simulations have illustrated the effect of the forming tool’s installation on the stresses occurring on the working surface of the nozzle. The curves shown in Figure 19b indicate that the presence of the tool leads to a significant increase in stress in certain areas of the nozzle, which directly contributes to greater wear of both the nozzle and the forming tools. In the configuration without forming tools, the stress curve on the working surface of the nozzle rises to 400 MPa before flattening out. However, when a tool is mounted at the end of the nozzle, the stress at this point reaches 500 Mpa before beginning to stabilize.

**Figure 21:**
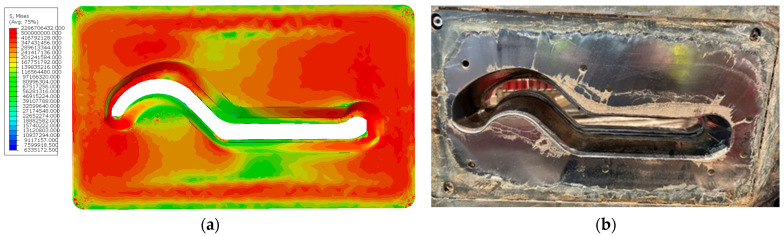
Numerical simulation results of band extrusion: (**a**) equivalent stress (von Mises), (**b**) forming tool during operation.

Figure 21 presents the numerical simulation results for the forming assembly (nozzle and forming tool) from inside the conical head chamber. At this stage, the compressed forming mass exerts pressure on these tools before passing through the opening that provides the initial shape to the produced tile. The results indicate that the highest stresses operate on the surface of the nozzle, where most areas experience stress exceeding 500 Mpa, with localized maxima of over 2000 Mpa. These high stresses in this region result from the pressure exerted by the entire forming mass filling the conical head chamber.

## 4. Conclusions

This study compiles the results of a comprehensive analysis of forming tools used in the production of ceramic tiles. The conducted research included an operational analysis of the forming tools, together with an examination of the forming material and the parameters influencing intensive wear. The presented results make it possible to point to the best approach for analyzing the extrusion process for ceramic tile bands and identifying the most effective methods for increasing tool durability. The key conclusions include:

- The key parameters influencing the wear of forming tools in the extrusion process are the extrusion pressure and the band speed. In the conducted operational tests, the extrusion pressure was 21 bar, and the band speed was 20 m/min.
- The primary plastic component of the forming mass is vermiculite. The mass also contains hard fractions such as quartz, zircon, garnet, and ceramic fragments, which significantly contribute to the abrasive wear of forming tools.
- Among the tested hardening variants of NC11LV steel, the best performance in operational tests was observed in the tool hardened at 1020 °C and tempered at 200 °C for 2 h.
- An additional confirmation of the operational test results was provided by the dry abrasion test, conducted on samples from hardened tools. The results were consistent with the operational tests, again showing that NC11LV steel hardened at 1020 °C and tempered at 200 °C for 2 h exhibited the least wear. This indicates that the dry abrasion test is a reliable method for evaluating forming tools, given the use of electrocorundum as the abrasive.
- The service life of the tool was further extended by nitriding the hardened NC11LV steel tool.
- The most significant wear in the operational tests was observed in the Hardox 600 steel tool with an additional overlay weld, likely due to the tempering of the base material caused by improper welding techniques.
- The dominant wear mechanisms for all the tested materials include surface scratching, while irregular deformations were observed on the working surface of the hardened NC11LV steel.
- The best method for gaining deeper insights into the ceramic tile band extrusion process is numerical modelling. Given the large deformations occurring in this technological process, the mesh-free SPH modelling approach is the most suitable solution.

## Figures and Tables

**Table 1:** Tool materials examined under operational conditions.

Test No.	Description of Forming Tool 1	Description of Forming Tool 2
Test 1	Steel NC11LV—hardening: 960 °C, tempering 450 °C for 2 h	Steel NC11LV—hardening: 1020 °C, tempering 200 °C for 2 h
Test 2	Steel NC11LV—hardening: 1060 °C, tempering 450 °C for 2 h	
Test 3	Steel NC11LV—hardening: 1020 °C, tempering 200 °C for 2 h + nitriding	
Test 4	Steel NC11LV—hardening: 1020 °C, tempering 200 °C for 2 h + nitriding	
Test 5	Steel Hardox 600—surfacing	

## Data Availability

The raw data supporting the conclusions of this article will be made available by the authors on request.
